# Gender differences in the association between changes in the atherogenic index of plasma and cardiometabolic diseases: a cohort study

**DOI:** 10.1186/s12944-024-02117-w

**Published:** 2024-05-07

**Authors:** Xingjie Huang, Song Wen, Yuqing Huang, Zehan Huang

**Affiliations:** 1 Department of Cardiology, The Second Affiliated Hospital of Guilin Medical University, Guilin, 541000 Guangxi China; 2 Department of Cardiology, Guangdong Cardiovascular Institute, Guangdong Provincial People’s Hospital, Guangdong Academy of Medical Sciences, Southern Medical University, Guangzhou, Guangdong 510080 China; 3 Hypertension Laboratory, Department of Cardiology, Guangdong Cardiovascular Institute, Guangdong Provincial People’s Hospital, Guangdong Academy of Medical Sciences, Southern Medical University, Guangzhou, Guangdong 510080 China

**Keywords:** Atherogenic index of plasma (AIP), Diabetes, Stroke, Cardiometabolic Diseases

## Abstract

**Objective:**

The relationship between changes in Atherogenic Index of Plasma (AIP) and cardiometabolic diseases (CMD) in middle-aged and elderly individuals remains unclear. This study aims to explore the association between changes in AIP and CMD.

**Methods:**

This study included 3,791 individuals aged over 45 years from CHARLS. Participants were divided into four groups using the K-Means clustering method. Cumulative AIP was used as a quantitative indicator reflecting changes in AIP. Differences in baseline data and CMD incidence rates among these four groups were compared. Multifactorial logistic regression models were used to assess the relationship between changes in AIP and CMD, and subgroup analysis and interaction tests were conducted to evaluate potential relationships between changes in AIP and CMD across different subgroups. Restricted cubic splines (RCS) were used to assess the dose-response relationship between cumulative AIP and CMD.

**Results:**

Changes in AIP were independently and positively associated with CMD. In males, the risk significantly increased in class4 compared to class1 (OR 1.75, 95%CI 1.12-2.73). In females, changes in AIP were not significantly associated with CMD. Cumulative AIP was positively correlated with CMD (OR 1.15, 95%CI 1.01-1.30), with significant gender differences in males (OR 1.29, 95%CI 1.07-1.55) and females (OR 1.03, 95%CI 0.87-1.23) (*p* for interaction = 0.042). In addition, a linear relationship was observed between cumulative AIP and CMD in male.

**Conclusion:**

Substantial changes in AIP may increase the risk of CMD in middle-aged and elderly Chinese males. Dynamic monitoring of AIP is of significant importance for the prevention and treatment of CMD.

**Supplementary Information:**

The online version contains supplementary material available at 10.1186/s12944-024-02117-w.

## Introduction

Cardiovascular Metabolic Diseases (CMD) encompass a range of conditions including diabetes, ischemic heart disease, stroke, and hypertension [1, 2]. With the global population aging and continuous lifestyle changes, the worldwide incidence of CMD is on the rise [3]. These diseases not only burden healthcare systems but also significantly impact patients' quality of life. Coronary heart disease, diabetes, and stroke, in particular, pose major threats to human health, with ischemic heart disease and stroke being the second and third leading causes of disability-adjusted life years globally in 2019 [4]. In China, CMD is a leading cause of death and disability [5]. The health impact is more significant when an individual has two or more CMDs, leading to worse long-term prognoses [6, 7]. With a deeper understanding of CMD, this condition has been defined as Cardiovascular Metabolic Multimorbidity (CMM) [8]. Although a precise definition of CMM has yet to reach consensus, the phenomenon is universally recognized [9–11]. Accumulating evidence from evidence-based medicine has identified several cardiac metabolic biomarkers related to CMD [12], among which the Atherogenic Index of Plasma (AIP) has garnered increasing attention [13].

AIP was proposed by Dobiásová and Frohlich in 2001 [14], calculated as the negative logarithm of the ratio of triglycerides (TG) to high-density lipoprotein cholesterol (HDL-C). AIP not only reflects lipid characteristics but is also closely associated with atherosclerosis, formation and progression of coronary artery plaques, acute coronary thrombosis, and chronic coronary occlusion [15–17]. Epidemiological studies have shown that AIP is closely related to insulin resistance (IR) and is an important predictor of hypertension, diabetes, obesity, and other metabolic syndromes [18–20]. An elevated AIP is also closely linked to an increased risk of adverse cardiovascular events [21]. As a simple and cost-effective biomarker, AIP can be widely used in clinical and epidemiological research to assess an individual's risk of cardiovascular disease. Therefore, monitoring and managing AIP could become a key indicator in the prevention and treatment of CMD.

Although existing studies have shown a positive relationship between single-time AIP levels and the risk of CMD [15, 22, 23], the relationship between cumulative elevation or improvement of AIP and CMD has not been fully clarified. While a few studies have explored the association between cumulative elevation of AIP and the incidence of new strokes [24], the impact of AIP changes on CMD requires further validation. Hence, this study aims to assess the impact of AIP changes on the incidence of CMD in middle-aged and elderly Chinese individuals, providing new strategies for the prevention and treatment of CMD in this population.

### Study population

This study was based on data from the China Health and Retirement Longitudinal Study (CHARLS), which is one of the few nationally representative prospective cohort studies on the health of middle-aged and elderly individuals in China [25]. The sample for CHARLS was obtained from 450 communities within 150 districts and 28 provinces through multistage probability sampling, and 10,257 households participated with 17,708 individuals in the baseline survey, with follow-ups every two years. The CHARLS project has been ethically reviewed by the Biomedical Ethics Committee of Peking University, and all participants have signed informed consent forms.

**Fig. 1:**
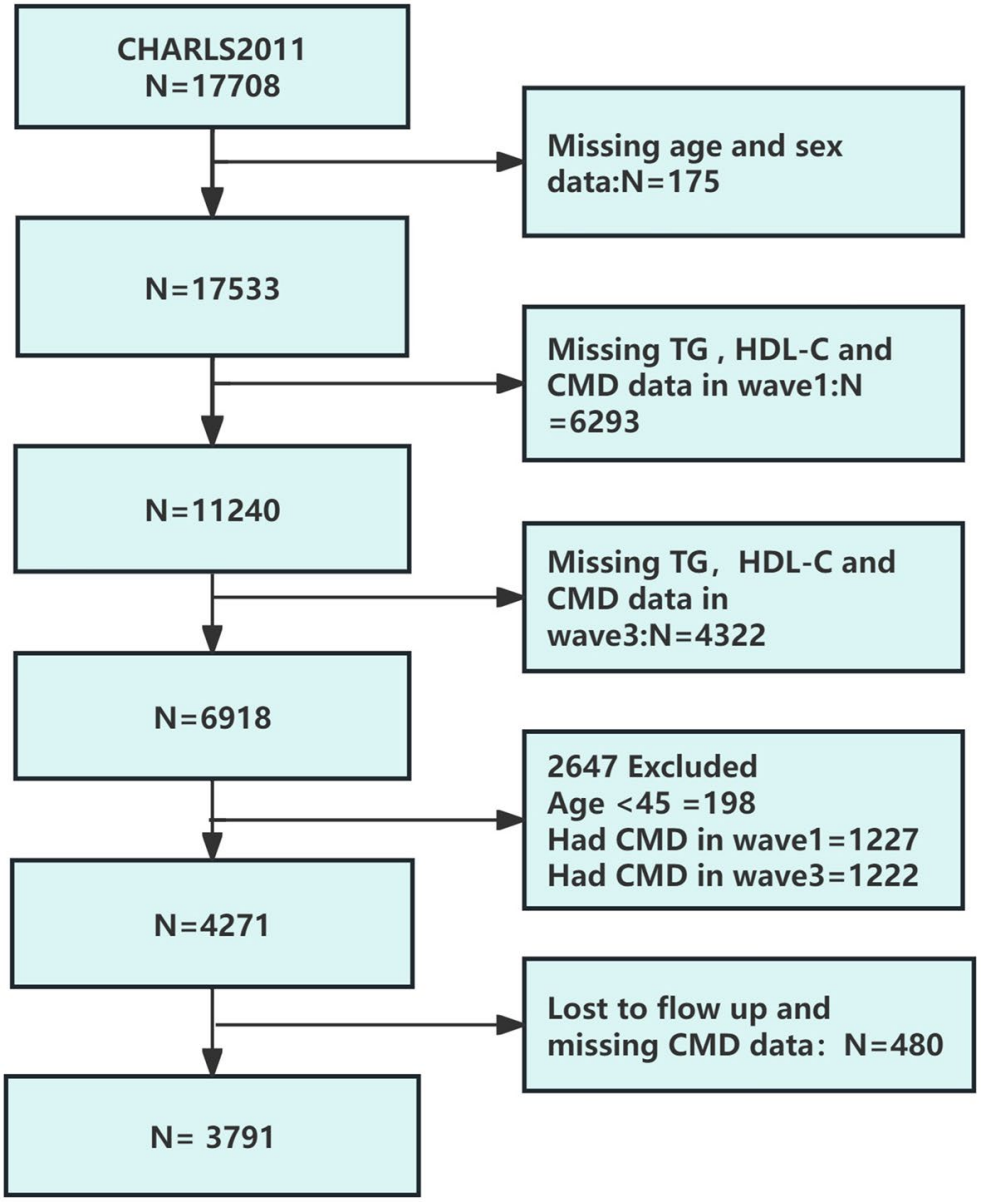
Study Population Flow Chart. CMD = Cardiometabolic Disease, HDL-C = High-Density Lipoprotein Cholesterol, TG = Triglyceride

Considering available blood examination data, the datasets for Wave 1 (baseline, 2011) and Wave 3 (follow-up, 2015) were extracted. We first included 17,708 participants at Wave 1. Exclusion criteria were: 1) no information about age and sex (n=175); 2) without complete data on triglyceride (TG), HDL-C or CMD at Wave 1 (n=6293) or Wave 3 (n=4,322); 3) persons aged less than 45 years old (n=198); 4) participants with established diagnosis of CMD at Wave 1 (n=1,227) or Wave 3 (n=1,222); 5) participants lost follow up and missing CMD data (n=480). Finally, 3,791 respondents completed two follow-ups in 2018 (Wave4) and 2020 (Wave5), with a follow-up period of about 5 years, and were included in this study for analysis (Fig. 1).

### Outcome variables

Diabetes was determined by previous doctor diagnosis, current use of glucose-lowering medication (including traditional Chinese medicine, Western medicine, insulin injections, etc.), or hemoglobin A1c levels ≥6.5%, fasting blood glucose ≥126mg/dL [26]. Stroke was determined based on the respondent's self-report, i.e., whether a doctor had informed them of a previous stroke occurrence [27]. Heart disease was determined by previous doctor diagnosis of heart disease (including myocardial infarction, coronary heart disease, angina, congestive heart failure), regardless of whether they were receiving related medication or other treatments [28]. The primary endpoint of this study was CMD, defined as the occurrence of any of diabetes, stroke, or heart disease during the follow-up period. CMM was defined as the occurrence of two or more CMDs during the follow-up period.

### Exposure variables

The primary exposure variable of this study was the change in AIP between 2012 and 2015, calculated as AIP = log10[TG (mg/dL)/HDL-C(mg/dL)]. Following the methods of previous studies [27, 29], we calculated the cumulative exposure of AIP, i.e., cumulative AIP = (AIP2012 + AIP2015)/2 × time (2015−2012).

### Covariables

The study collected participants' demographic characteristics, past health status, and lifestyle information through questionnaires. In this study, we categorized and defined certain variables. Education level was divided into "junior high school and below (low level)" and "above junior high school (high level)"; marital status was divided into "married/cohabiting" and "divorced/widowed/separated/single"; residence was divided into "rural" and "urban". Lifestyle surveys included smoking and drinking behaviors, with smoking behavior divided into "yes" and "no", representing "never smoked" and "past or current smoker", respectively; drinking behavior was also divided into "yes" and "no", representing "never drank" and "past or current drinker".

Dyslipidemia was defined as a previous doctor diagnosis or current use of lipid-lowering medication [30]. Hypertension was defined as a self-report of a previous doctor diagnosis of hypertension, current use of antihypertensive medication, systolic blood pressure ≥140 mmHg, or diastolic blood pressure ≥90 mmHg [31]. Height and weight were measured by professional technicians, and BMI was calculated as weight (kg) divided by the square of height (m). Our research reflects an individual's economic level through per capita annual household consumption, where Per capita consumption = total household consumption / number of people living in this household (according to the tertile, it is divided into high, medium and low levels).

In 2012 and 2015, respondents had venous blood samples collected by professional technicians after fasting for over 8 hours, for testing total cholesterol (TC), high-density lipoprotein cholesterol (HDL-C), low-density lipoprotein cholesterol (LDL-C), triglycerides (TG), fasting blood glucose (GLU), glycated hemoglobin A1c (HbA1c), uric acid (UA), creatinine (Cr), blood urea nitrogen (BUN), and C-reactive protein (CRP) among other indicators.

### Statistical analysis

**Fig. 2:**
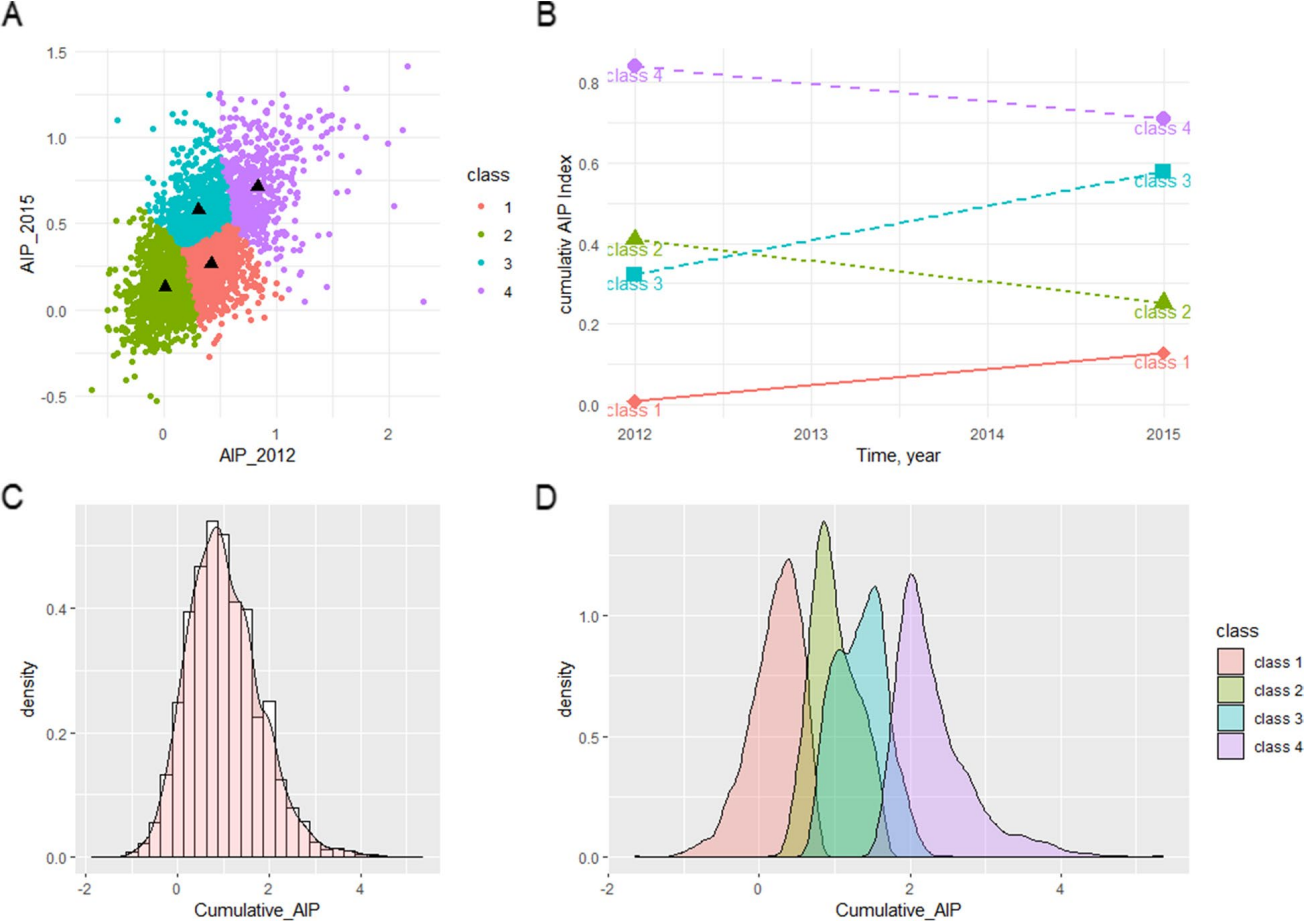
**A** Clustering diagrams for AIP2012 and AIP2015; **B** Grouping diagram after k-means clustering; **C** Histogram and probability density plot of cumulative AIP in the overall population, illustrating the data distribution of cumulative AIP. **D** Histograms and probability density plots of cumulative AIP for groups class1-4, showing the data distribution of cumulative AIP within these four groups. AIP = Atherogenic Index of Plasma

In this study, we utilized the "cluster" and "factoextra" packages to perform K-means cluster analysis on AIP measurements from 2012 and 2015 [32], aiming to provide a basis for grouping study subjects. By applying the elbow method, we identified four sample centers, subsequently assigning samples to the nearest cluster and updating cluster center points. This process was repeated until cluster center points stabilized or a predetermined number of iterations was reached. Ultimately, we obtained four cluster center points for 2012 and 2015 (0.323, 0.007, 0.409, 0.841 and 0.577, 0.129, 0.253, 0.709, respectively). Based on clinical situations and cluster center values, we divided study subjects into four groups: "persistently low level" (class1), "median level decrease" (class2), "median level increase" (class3), and "persistently high level" (class4) (Fig. 2). When statistically describing the basic characteristics of these four groups of study subjects, categorical variables were presented as frequency (n) and percentage (%), and differences between groups were compared using the chi-square test. For continuous variables that followed a normal distribution, we described them using mean ± standard deviation and used one-way ANOVA for between-group comparisons; for continuous variables that did not follow a normal distribution, we presented them as median (interquartile range) M(Q1,Q3) and used the Kruskal-Wallis test for between-group comparisons. Additionally, we constructed three multivariable logistic regression models to assess the relationship between AIP changes and CMD, where model 1 did not adjust for any variables, model 2 adjusted for age and gender, and model 3 further adjusted for multiple variables including education, current marital status, residence, consumption, smoking, drinking, BMI, SBP, dyslipidemia, hypertension, TC, LDL-C, GLU, HbA1c, CRP, Cr, BUN, and UA. Trend tests were used to assess the trend relationship between AIP changes and CMD, and subgroup analysis was used to explore the potential relationship between AIP changes and CMD under different stratifications such as age, gender, smoking, drinking BMI, rural household registration, hypertension, education, and consumption. Restricted cubic splines (RCS) were used to assess whether there was a nonlinear association between cumulative AIP and CMD [Based on the principle of minimizing the AIC (Akaike Information Criterion) and the distribution of cumulative AIP data, we ultimately selected 3 knots, namely at the 10th percentile (P10), the 50th percentile (P50), and the 90th percentile (P90)]. Sensitivity analysis was used to assess the stability of the relationship between AIP changes and CMD after handling missing values. We used the "mice" package for multiple imputation of missing variables and the "MatchIt" package for propensity score matching (PSM) to balance baseline data.

Table s1 displayed the distribution of missing data variables in our study. To maintain as large a sample size as possible, we employed multiple imputation methods to handle missing variables for sensitivity analysis. During the data cleaning process, we detected outliers in cumulative AIP using the 3σ rule, defining any cumulative AIP < mean -3SD or cumulative AIP > mean +3SD as outliers, identifying a total of 31 outliers. In the sensitivity analysis section, we excluded these outliers before conducting further analysis to assess the stability of the results.

In this study, a two-sided p-value of less than 0.05 was defined as statistically significant. All statistical analyses were performed using Stata software (version 18.0, StataCorp) and R software (version 4.2.2, http://www.R-project.org, The R Foundation).

## Results

### Intra-group comparison and distribution of AIP-related indicators

Paired t-tests were conducted on AIP values from 2012 and 2015, revealing significant changes across all categories: class1 (0.01±0.14 vs. 0.13±0.16, *p* < 0.001), class2 (0.41±0.15 vs. 0.25±0.12, *p* < 0.001), class3 (0.32±0.16 vs. 0.58±0.14, *p* < 0.001), class4 (0.84±0.25 vs. 0.71±0.22, *p* < 0.001) (supplementary figure). Histograms and probability density plots indicated that cumulative AIP, AIP2012, and AIP2015 exhibited characteristics of a normal distribution in the overall population and in each subgroup (Fig. 2, supplementary figure).

### Baseline data comparison

A total of 3,791 participants were included in the analysis, with 47% male (1,773) and 53% female (2,018), and an average age of 57.41±8.37 years. The mean value of cumulative AIP was 1.01±0.80. By the end of the follow-up period, 918 participants (24%) had developed CMD. In class4, levels of hypertension, dyslipidemia, BMI, SBP, DBP, consumption, TC, LDL-C, GLU, UA, TG2012, TG2015, AIP2012, and AIP2015 were significantly elevated. In contrast, age, male proportion, residence (rural), drinking, and levels of HDL-C2012, HDL-C2015, and BUN, were lower in class4. There were no significant differences in education, current marital status, smoking, HbA1c, CRP and Cr across the four groups. Cumulative AIP increased progressively from class1 to class4 (0.21±0.34 vs. 0.99±0.30 vs. 1.35±0.33 vs. 2.32±0.53, *p* < 0.001), as did the incidence of CMD (20% vs. 25% vs. 26% vs. 29%, *p* < 0.001) (Table 1).

**Table 1:** Baseline characteristics of participants according to the change in the AIP

Characteristic	Overall, *N* = 3,791	class 1, *N* = 1,266	class 2, *N* = 1,090	class 3, *N* = 861	class 4, *N* = 574	*p*-value
Age(year)	57.41±8.37	58.24±8.60	57.68±8.29	56.62±8.36	56.26±7.80	<0.001
Sex(male)	1,773 (47%)	626 (49%)	525 (48%)	372 (43%)	250 (44%)	0.010
Education						0.121
Low level	3,456 (91%)	1,171 (92%)	995 (91%)	776 (90%)	514 (90%)	
High level	335 (8.8%)	95 (7.5%)	95 (8.7%)	85 (9.9%)	60 (10%)	
Current marital status						
Married/cohabiting	3,467 (91%)	1,144 (90%)	992 (91%)	792 (92%)	539 (94%)	0.075
Divorced/widowed/separated/single	324 (8.5%)	122 (9.6%)	98 (9.0%)	69 (8.0%)	35 (6.1%)	
Residence (rural)	2,596 (68%)	940 (74%)	735 (67%)	582 (68%)	339 (59%)	<0.001
Consumption (ten thousand yuan per year)	0.46 (0.27, 0.79)	0.44 (0.27, 0.74)	0.47 (0.28, 0.85)	0.44 (0.27, 0.78)	0.50 (0.29, 0.88)	0.017
Drinking	1,491 (39%)	542 (43%)	419 (38%)	308 (36%)	222 (39%)	0.009
Smoking	1,460 (39%)	513 (41%)	426 (39%)	312 (36%)	209 (36%)	0.153
Hypertension	1,174 (34%)	306 (27%)	334 (34%)	287 (37%)	247 (47%)	<0.001
Dyslipidemia	228 (6.0%)	40 (3.2%)	68 (6.2%)	56 (6.5%)	64 (11%)	<0.001
BMI (kg/m2)	23.14±3.69	21.82±3.19	23.14±3.62	23.86±3.72	25.00±3.72	<0.001
SBP (mmHg)	126.28±20.06	123.11±18.93	126.83±19.54	127.16±21.08	130.99±20.79	<0.001
DBP (mmHg)	74.20±11.85	71.95±10.99	74.20±11.76	75.37±12.10	77.49±12.52	<0.001
TC (mg/dl)	191.16±37.93	187.27±32.85	187.73±37.63	194.37±37.98	201.45±45.85	<0.001
LDL-C (mg/dl)	114.94±33.49	111.71±28.21	115.51±33.28	124.45±34.20	106.62±39.73	<0.001
GLU (mg/dl)	102.85±19.66	100.17±15.18	103.15±22.05	101.11±18.02	110.82±23.47	<0.001
HbA1c (%)	5.09±0.43	5.08±0.44	5.10±0.47	5.09±0.38	5.08±0.43	0.823
CRP (mg/dl)	2.27±6.78	2.17±7.53	2.04±4.77	2.35±6.29	2.79±8.71	0.169
BUN (mg/dl)	15.70±4.38	16.26±4.66	15.16±4.08	15.66±4.29	15.50±4.30	<0.001
Cr (mg/dl)	0.76±0.18	0.76±0.18	0.76±0.18	0.76±0.17	0.78±0.18	0.190
UA (mg/dl)	4.34±1.20	4.18±1.12	4.32±1.20	4.33±1.18	4.76±1.29	<0.001
TG 2012 (mg/dl)	100.00 (71.68, 144.26)	64.61 (53.99, 77.88)	117.71 (95.58, 146.02)	106.20 (83.19, 129.21)	219.48 (181.43, 290.28)	<0.001
TG2015 (mg/dl)	108.85 (79.65, 158.41)	78.76 (63.72, 96.46)	93.81 (78.76, 112.39)	164.60 (138.94, 204.42)	214.60 (163.72, 293.36)	<0.001
HDL-C 2012 (mg/dl)	52.10±14.94	64.49±13.73	47.96±10.04	50.01±10.13	35.77±8.29	<0.001
HDL-C 2015 (mg/dl)	52.32±11.80	59.90±12.28	52.87±9.37	46.22±7.84	43.68±8.36	<0.001
AIP 2012	0.32±0.32	0.01±0.14	0.41±0.15	0.32±0.16	0.84±0.25	<0.001
AIP 2015	0.35±0.27	0.13±0.16	0.25±0.12	0.58±0.14	0.71±0.22	<0.001
Cumulative AIP	1.01±0.80	0.21±0.34	0.99±0.30	1.35±0.33	2.32±0.53	<0.001
CMD	918 (24%)	258 (20%)	268 (25%)	228 (26%)	164 (29%)	<0.001

Continuous variables are expressed as mean ± standard deviation or interquartile range, and categorical variables as frequencies (n) and percentages (%)

*BMI* Body Mass Index, *TC* Total Cholesterol, *TG* Triglyceride, *LDL-C* Low-Density Lipoprotein Cholesterol, *HDL-C* High-Density Lipoprotein Cholesterol, *UA* Uric Acid, *GLU* Glucose, *Cr* Creatinine, *BUN* Bilirubin, *HbA1c* Hemoglobin A1c, *CRP* C-Reactive Protein, *SBP* Systolic Blood Pressure, *DBP* Diastolic Blood Pressure, *AIP* Atherogenic Index of Plasma

### CMD risk analysis

Multivariable logistic regression analysis of CMD risk revealed that compared to class1, the risk in class4 was significantly higher (OR 1.40, 95%CI 1.04-1.88, *p* = 0.026), as was the risk in class3 (OR 1.34, 95%CI 1.04-1.71, *p* =0.023), and class2 (OR 1.31, 95%CI 1.04-1.66, *p* = 0.021). Trend tests indicated a significant increase in CMD incidence with rising AIP levels (*p* < 0.05). When cumulative AIP was introduced as a continuous variable into the multivariate regression model, the results in model 3 were significant (OR 1.15, 95%CI 1.01-1.30, *p* = 0.031). Additionally, when cumulative AIP was stratified into quartiles, the risk of CMD in the third quartile relative to the first quartile was significantly elevated in model 3 (OR 1.41, 95% CI 1.09-1.83, *p* = 0.010). In contrast, the increase in CMD risk in the fourth quartile was higher (OR 1.28, 95% CI 0.98-1.69, *p* = 0.070) but did not reach statistical significance. Trend tests also showed a significant increase in CMD incidence with higher cumulative AIP (*p* < 0.05) (Table 2).

**Table 2:** Associations between different classes of the AIP and incidence of CMD

	**CMD, N(%)**	**Model 1**			**Model 2**			**Model 3**		
		***OR***	***95% CI***	*p*-value	***OR***	***95% CI***	*p*-value	***OR***	***95% CI***	*p*-value
**Categories**										
** Class 1**	258 (20%)	—	—		—	—		—	—	
** Class 2**	268 (25%)	1.27	(1.05-1.55)	0.014	1.30	(1.07-1.58)	0.009	1.31	(1.04-1.66)	0.021
** Class 3**	228 (26%)	1.41	(1.15- 1.73)	0.001	1.47	(1.20-1.81)	<0.001	1.34	(1.04-1.71)	0.023
** Class 4**	164 (29%)	1.56	(1.24-1.96)	<0.001	1.65	(1.31- 2.08)	<0.001	1.40	(1.04-1.88)	0.026
*** P for trend***				<0.001			<0.001			0.030
** Cumulative AIP**		1.22	(1.11-1.34)	<0.001	1.25	(1.14-1.37)	<0.001	1.15	(1.01-1.30)	0.031
**Categories**										
** Q1(-1.65,0.449)**	188 (20%)	—	—		—	—		—	—	
** Q2(0.449,0.937)**	216 (23%)	1.18	(0.95- 1.47)	0.137	1.20	(0.96-1.49)	0.114	1.13	(0.87-1.47)	0.361
** Q3(0.937,1.49)**	251 (27%)	1.51	(1.22-1.88)	<0.001	1.56	(1.26-1.94)	<0.001	1.41	(1.09-1.83)	0.010
** Q4(1.49,5.36)**	263 (27%)	1.52	(1.23-1.88)	<0.001	1.60	(1.29-1.99)	<0.001	1.28	(0.98-1.69)	0.070
***P for trend***				<0.001			<0.001			0.026

Results are presented as Odds Ratios (OR) with 95% Confidence Intervals (CI)

*BMI* Body Mass Index, *CI* Confidence Interval, *TC* Total Cholesterol, *TG* Triglyceride, *HDL-C* High-Density Lipoprotein Cholesterol, *LDL-C* Low-Density Lipoprotein Cholesterol, *UA* Uric Acid, *GLU* Glucose, *Cr* Creatinine, *BUN* Bilirubin, *HbA1c* Hemoglobin A1c, *CRP* C-Reactive Protein, *SBP* Systolic Blood Pressure, *DBP* Diastolic Blood Pressure, *AIP* Atherogenic Index of Plasma, *OR* Odds Ratio

Model 1: Unadjusted variables

Model 2: Adjusted for age, and gender

Model 3: In addition to the variables adjusted in Model 2 (age, gender), the following variables were added: education, current marital status, residence, consumption, smoking, drinking, BMI, SBP, hypertension, dyslipidemia, TC, LDL-C, GLU, HbA1c, CRP, Cr, BUN, UA

### CMM risk analysis

Further analysis of CMM risk using multivariable logistic regression showed that compared to class1, the risk in class4 was significantly higher in model 3 (OR 3.09, 95%CI 1.56-6.23, *p* = 0.001), while the risks in class3 (OR 1.38, 95%CI 0.70-2.76, *p* =0.347) and class2 (OR 1.62, 95%CI 0.87-3.10, *p* = 0.136) were elevated but not statistically significant. Trend tests revealed a significant increase in CMM incidence with higher AIP levels (*p* = 0.003). When cumulative AIP was introduced as a continuous variable into the multivariate regression model, model 3 results indicated a significant increase in CMM risk (OR 1.47, 95%CI 1.08-2.00, *p* = 0.013). After categorizing cumulative AIP into quartiles, the risk of CMM in the fourth quartile compared to the first was not significantly higher in model 3 (OR 1.77, 95%CI 0.91-3.61, *p* = 0.101). Trend tests also indicated a significant rise in CMM incidence with increasing cumulative AIP (*p* = 0.053) (Table 3).

**Table 3:** Associations between different classes of the AIP and incidence of CMM

	**CMM,** **N(%)**	**Model 1**			**Model 2**			**Model 3**		
		***OR***	***95% CI***	*p***-value**	***OR***	***95% CI***	*p***-value**	***OR***	***95% CI***	*p***-value**
**Categories**										
** Class 1**	23 (1.8%)	—	—		—	—		—	—	
** Class 2**	35 (3.3%)	1.63	(0.98-2.74)	0.061	1.56	(0.92-2.70)	0.103	1.62	(0.87-3.10)	0.136
** Class 3**	30 (3.6%)	1.90	(1.13-3.23)	0.016	1.81	(1.05-3.17)	0.033	1.38	(0.70-2.76)	0.347
** Class 4**	33 (5.9%)	2.91	(1.73-4.95)	<0.001	2.87	(1.65-5.05)	<0.001	3.09	(1.56-6.23)	0.001
***P for trend***				<0.001			<0.001			0.003
** Cumulative AIP**		1.43	(1.16-1.75)	<0.001	1.49	(1.21-1.82)	<0.001	1.47	(1.08-2.00)	0.013
**Categories**										
** Q1(-1.65,0.449)**	17 (1.8%)	—	—		—	—		—	—	
** Q2(0.449,0.937)**	23 (2.4%)	1.15	(0.62-2.12)	0.661	0.91	(0.48-1.72)	0.767	1.07	(0.51-2.24)	0.865
** Q3(0.937,1.49)**	37 (4.1%)	2.00	(1.17-3.52)	0.014	1.78	(1.02-3.17)	0.046	1.62	(0.83-3.29)	0.165
** Q4(1.49,5.36)**	44 (4.7%)	2.38	(1.42- 4.14)	0.001	2.07	(1.21-3.68)	0.010	1.77	(0.91-3.61)	0.101
***P for trend***				<0.001			<0.001			0.053

Results are presented as Odds Ratios (OR) with 95% Confidence Intervals (CI)

Abbreviations as in Table 2

Model 1: Unadjusted variables

Model 2: Adjusted for age, and gender

Model 3: In addition to the variables adjusted in Model 2 (age, gender), the following variables were added: education, current marital status, residence, consumption, smoking, drinking, BMI, SBP, hypertension, dyslipidemia, TC, LDL-C, GLU, HbA1c, CRP, Cr, BUN, UA

### Subgroup analysis results

**Fig. 3:**
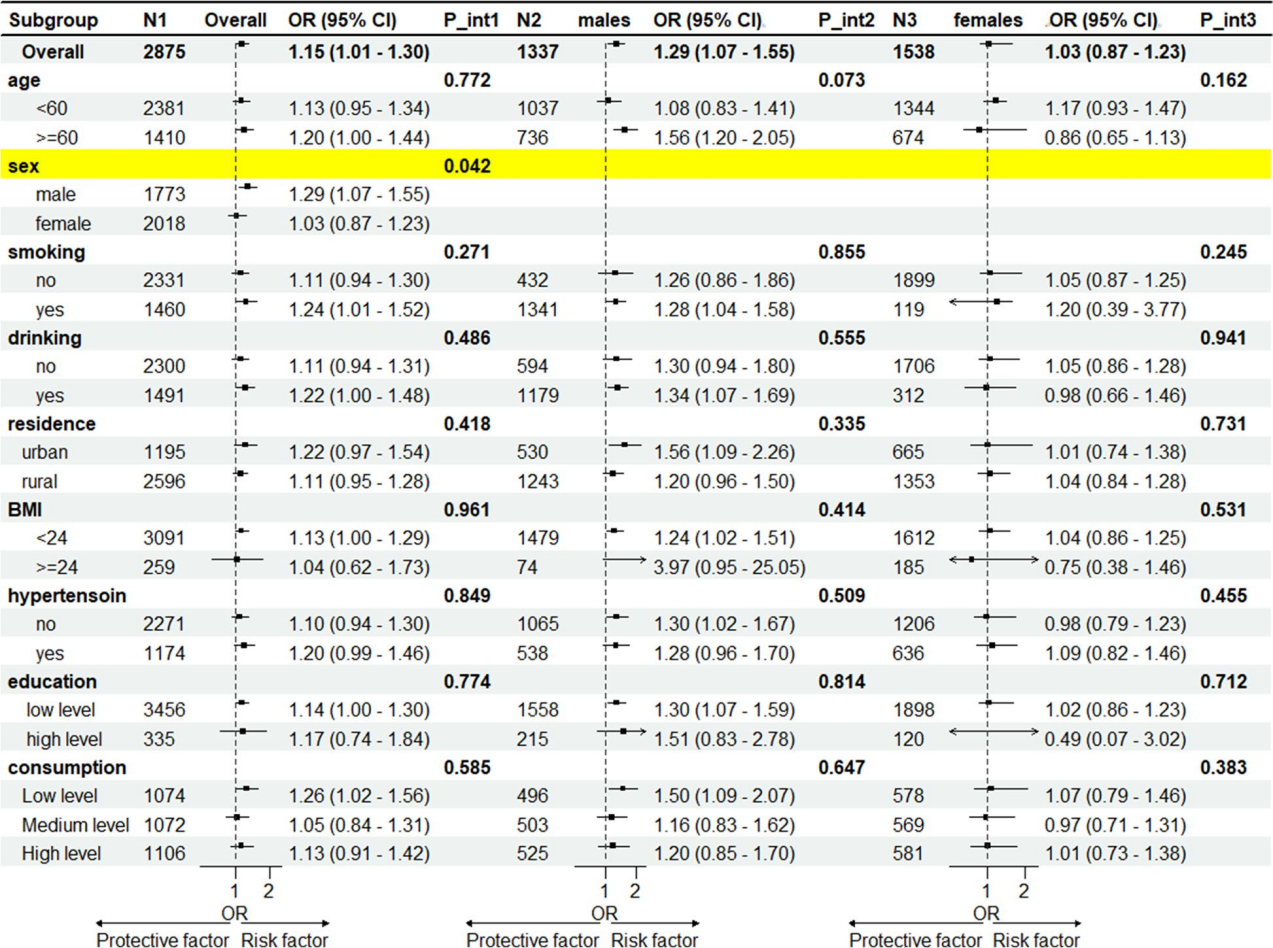
Subgroup and Interaction Analyses on the Association Between cumulative AIP and CMD. Results are presented as OR (95% CI). Adjustments were made for age, gender, education, current marital status, residence, consumption, BMI, smoking, drinking, dyslipidemia, hypertension, TC, LDL-C, GLU, HbA1c, CRP, Cr, BUN, UA, and SBP in the multivariable analysis model, excluding the strata variables. BMI = Body Mass Index, CI = Confidence Interval, TC = Total Cholesterol, LDL-C = Low-Density Lipoprotein Cholesterol, UA = Uric Acid, GLU = Glucose, Cr = Creatinine, BUN = Bilirubin, HbA1c = Hemoglobin A1c, CRP = C-Reactive Protein, AIP = Atherogenic Index of Plasma,P_int1= p for interaction of overall; P_int2 : p for interaction of males;P_int3: p for interaction of females

Subgroup analysis revealed that the association between changes in AIP and CMD was consistent overall. However, in gender-stratified subgroups, the change in AIP was significant for the male subgroup, particularly in class4 compared to class1 (OR 1.75, 95%CI 1.12-2.73, *p* = 0.035), class3 compared to class1 (OR 1.46, 95%CI 1.00- 2.15, *p* = 0.015), and class2 compared to class1 (OR 1.48, 95%CI 1.05-2.09, *p* = 0.013), while the association was not significant in the female subgroup. In the male subgroup, cumulative AIP was an independent predictor of CMD (OR 1.29, 95%CI 1.07-1.55, *p* = 0.008), but not in the female subgroup (OR 1.03, 95%CI 0.87-1.23, *p* = 0.704), with a statistically significant difference between genders (*p* for interaction = 0.042). Further gender-stratified subgroup analysis revealed that the relationship between cumulative AIP and CMD was significant in all subgroups among men, but not always significant among women (Fig. 3, Table 4).

**Table 4:** Associations of diferent classes of change in AIP with CMD incidence stratifed by diferent factors

Subgroups	N	Class 1	Class 2	Class 3	Class 4	*P for trend*	*P for ineraction*
			*OR(95% CI)*	*OR(95% CI)*	*OR(95% CI)*		
Age							0.744
<60	2208	ref	1.11(0.78-1.58)	1.33(0.91-1.95)	1.25(0.78-1.99)	0.041	
>=60	1583	ref	1.52(1.11-2.09)	1.38(0.99-1.94)	1.57(1.06-2.31)	0.013	
Sex							0.423
male	1773	ref	1.48(1.05-2.09)	1.46(1.00- 2.15)	1.75(1.12-2.73)	0.018	
female	2018	ref	1.16(0.88-1.66)	1.21(0.87-1.68)	1.18(0.78-1.76)	0.438	
Smoking							0.662
yes	1460	ref	1.60(1.08-2.36)	1.72(1.12-2.62)	1.59(0.97- 2.59)	0.063	
no	2331	ref	1.19(0.89-1.59)	1.17(0.86-1.60)	1.33(0.91-1.93)	0.155	
Drinking							0.390
yes	1491	ref	1.48(1.01-2.15)	1.24(0.82-1.87)	1.78(1.11-2.86)	0.038	
no	2300	ref	1.23(0.91-1.67)	1.39(1.01-1.91)	1.22(0.82-1.80)	0.242	
Residence							0.747
rural	2596	ref	1.33(1.01-1.74)	1.26(0.94-1.69)	1.24(0.85-1.79)	0.043	
urban	1195	ref	1.34(0.84-2.15)	1.56(0.96-2.55)	1.67(0.99-2.84)	0.313	
BMI							0.328
<24	3091	ref	1.33(1.05-1.69)	1.38(1.06-1.78)	1.30(0.94-1.78)	0.107	
>=24	259	ref	0.79(0.26-2.48)	0.64(0.20-2.08)	1.11(0.36-3.63)	0.953	
Hypertension							0.848
yes	1174	ref	1.41(0.94-2.10)	1.49(0.98-2.28)	1.42(0.89- 2.26)	0.136	
no	2271	ref	1.25(0.93-1.67)	1.25(0.91-1.71)	1.39(0.93-2.05)	0.112	
Education							0.816
low level	3456	ref	1.31(1.03-1.67)	1.34(1.03-1.74)	1.35(0.99-1.85)	0.058	
high level	335	ref	1.20(0.46-3.18)	1.29(0.46-3.63)	1.99(0.68-5.92)	0.217	
Consumption							0.585
low level	1074	ref	1.77(1.19-2.64)	1.58(1.03-2.41)	1.70(1.00-2.87)	0.042	
medium level	1072	ref	1.16(0.77-1.74)	1.18(0.76-1.82)	1.28(0.75-2.18)	0.360	
high level	1106	ref	1.13(0.74-1.73)	1.22(0.77-1.94)	1.24(0.75-2.05)	0.403	

Adjustments were made for age, gender, education, current marital status, residence, consumption, smoking, drinking, BMI, SBP, hypertension, dyslipidemia, TC, LDL-C, GLU, HbA1c, CRP, Cr, BUN, UA in the multivariable model, excluding the strata variables

Results are presented as Odds Ratios (OR) with 95% Confidence Intervals (CI)

Abbreviations as in Table 2

### Dose-response relationship analysis between cumulative AIP and CMD

**Fig. 4:**
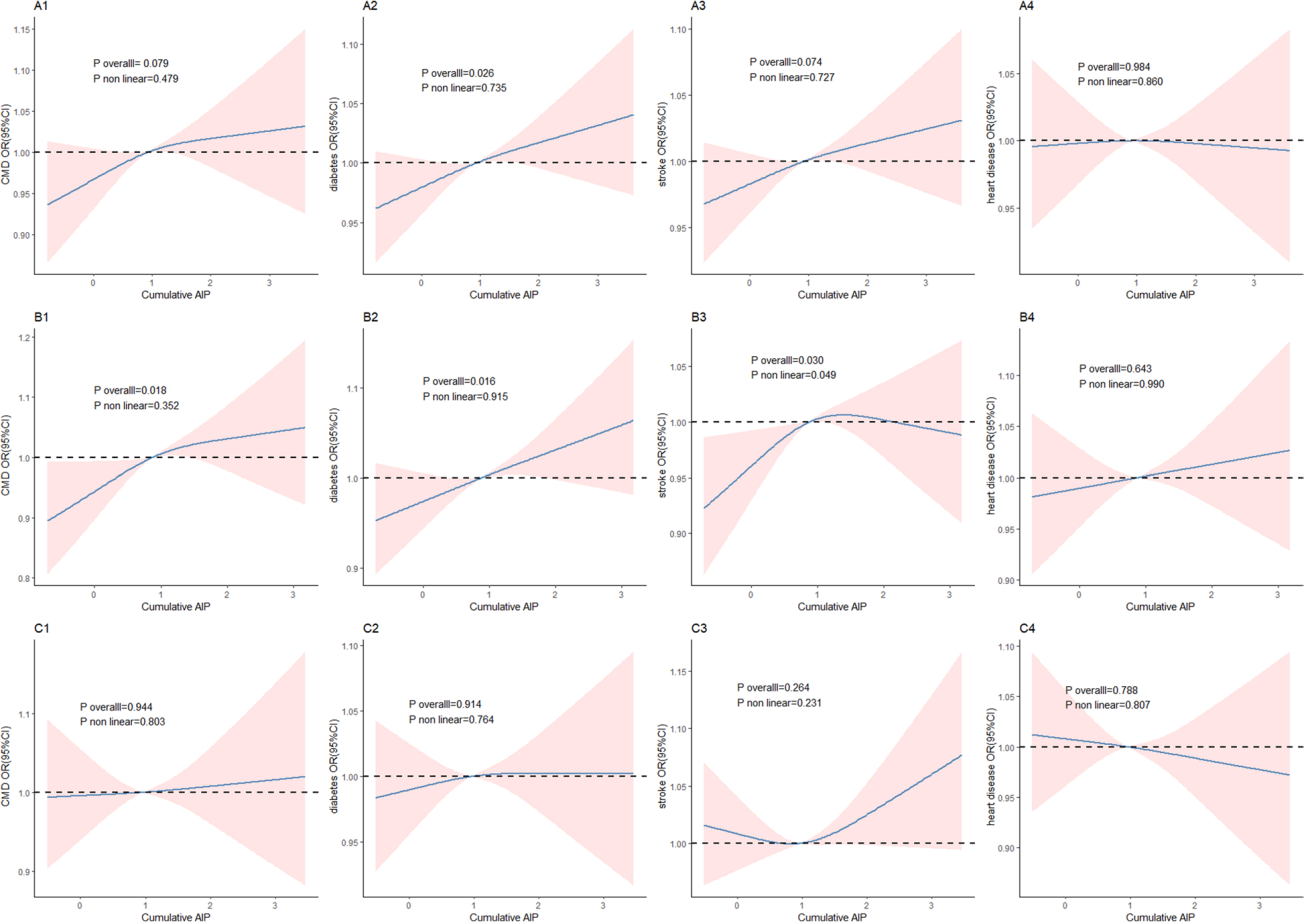
Dose-Response Curves of Cumulative AIP and CMD. Results are presented as OR (95% CI). A1-4: Represent the RCS curves of cumulative AIP with CMD, diabetes, stroke, and heart disease in the overall population. B1-4: Represent the RCS curves of cumulative AIP with CMD, diabetes, stroke, and heart disease in the male subgroup. C1-4: Represent the RCS curves of cumulative AIP with CMD, diabetes, stroke, and heart disease in the female subgroup. Adjustments were made for age, gender, education, current marital status, residence, consumption, BMI, smoking, drinking, dyslipidemia, hypertension, TC, LDL-C, GLU, HbA1c, CRP, Cr, BUN, UA, and SBP,in the RCS model, excluding the strata variables. BMI = Body Mass Index, CI = Confidence Interval, TC = Total Cholesterol, TG = Triglyceride, HDL-C = High-Density Lipoprotein Cholesterol, LDL-C = Low-Density Lipoprotein Cholesterol, UA = Uric Acid, GLU = Glucose, Cr = Creatinine, BUN = Bilirubin, HbA1c = Hemoglobin A1c, CRP = C-Reactive Protein, SBP = Systolic Blood Pressure, AIP = Atherogenic Index of Plasma, OR = Odds Ratio

In the male subgroup, cumulative AIP is linearly associated with CMD (*p* for non-linearity = 0.352) and diabetes (*p* for non-linearity = 0.915), exhibits a non-linear relationship with stroke (*p* for non-linearity = 0.049), and has no significant association with heart disease. In the overall population, cumulative AIP has a linear relationship with CMD (p for non-linearity = 0.735), but its association with CMD, stroke, and heart disease is not significant. In the female subgroup, the relationship between cumulative AIP and CMD, as well as its sub-endpoints, is not significant (Fig. 4).

### Sensitivity analysis

To ensure the stability and reliability of the study results, various sensitivity analysis methods were employed to assess the potential impact of missing data on the study conclusions. First, the 3,791 participants were divided into a complete data group and a missing value group, and the baseline conditions of the two groups were compared. The comparison showed that although the participants in the missing value group were younger on average, there were no significant differences between the two groups in terms of gender distribution, cumulative AIP levels (Table S1). Further, variables with missing values were excluded from model 3, which did not affect the study's main findings, with the comparison between class4 and class1 (OR 1.46, 95%CI 1.13-1.86, *p* = 0.002) (Table S2b) still showing a significant association between cumulative AIP and CMD (OR 1.19, 95%CI 1.07-1.31, *p* < 0.001) (Table S2a), as well as the comparison between the fourth and first quartiles (OR 1.44, 95%CI 1.16-1.82, *p* = 0.001) (Table S2b). Subsequently, we excluded the outliers before proceeding with the analysis,with the comparison between class4 and class1 (OR 1.39, 95%CI 1.03-1.87, *p* = 0.029) (Table S3b) still showing a significant association between cumulative AIP and CMD (OR 1.14, 95%CI 1.01-1.29, *p* = 0.043) (Table S3a), as well as the comparison between the fourth and first quartiles (OR 1.28, 95%CI 0.97-1.68, *p* = 0.076) (Table S3b). Additionally, the propensity score matching (PSM) method was used to divide participants into two groups (Q1-2 and Q3-4) based on the median cumulative AIP, and matched at a 1:1 ratio using the nearest neighbor matching method with a caliper value of 0.2, successfully matching 1,036 pairs of samples. Logistic regression analysis conducted again showed that the comparison between class4 and class1 (OR 1.52, 95%CI 1.09-2.13, *p* = 0.014) (Table S2b) and the significant relationship between cumulative AIP and CMD (OR 1.18, 95%CI 1.03-1.36, *p* = 0.016) (Table S2a) remained, as well as the comparison between the higher and lower quartiles (OR, 1.21 95%CI 1.00-1.49, *p* = 0.050) (Table S2b). Finally, multiple imputation was used to handle missing data, generating five complete datasets for analysis. The combined regression analysis results from these datasets confirmed the comparison between class4 and class1 (OR 1.34, 95%CI 1.04-1.74, *p* = 0.026) (Table S3b), the significant relationship between cumulative AIP and CMD (OR 1.15, 95%CI 1.03-1.29, *p* = 0.010) (Table S3a), and the comparison between the fourth and first quartiles (OR 1.29, 95%CI 1.02-1.63, *p* = 0.035) (Table S3b). These sensitivity analyses allow us to confidently assert that the study's conclusions are robust and not significantly affected by missing data.

## Discussion

This study analyzed 5-year follow-up data of 3,791 middle-aged and elderly Chinese individuals and found that: 1) 918 individuals (24%) developed CMD by the end of the follow-up; 2) Middle-aged and elderly individuals with persistently high AIP levels, or those whose levels increased or decreased from the median, had a significantly higher risk of CMD than those with persistently low AIP levels; 3) Higher AIP levels, when effectively controlled, could reduce the risk of CMD to some extent. In 2012, the AIP level of class2 was significantly higher than that of class3, while in 2015, the opposite was true. Multivariable regression analysis showed that the risk of CMD in class2 was 1.31 times that of class1, and in class3, it was 1.34 times; 4) Persistently high AIP levels were independent predictors of CMM risk in the middle-aged and elderly; 5) The relationship between changes in AIP and cumulative AIP with CMD was more significant in men than in women, with a significant interaction effect between cumulative AIP and gender (*p* for interaction = 0.042); 6) The dose-response curve between cumulative AIP and CMD showed a linear relationship in male.

This study explored the relationship between dynamic changes in AIP and CMD risk and found conclusions similar to previous studies. Song et al. [33] reported that for every standard deviation increase in AIP, the risk of diabetes increased by 0.45 times. A meta-analysis [23] also confirmed that with increasing AIP, levels of IR and the risk of T2DM increased. Although Yi et al. [34] analyzed the longitudinal change in AIP and the risk of diabetes in middle-aged and elderly individuals, with conclusions similar to this study, it was found that including newly diagnosed diabetes in wave2 and wave3 as outcome variables may have affected the accuracy of assessing the causal relationship between changes in AIP and newly diagnosed diabetes. In our study, only subjects with newly diagnosed CMD in wave4 and wave5 were included in the outcome variables, resulting in a clearer causal relationship. Additionally, we used a machine learning method (k-means clustering) for an objective and scientific classification of changes in AIP. In our study, only subjects with newly diagnosed CMD in wave4 and wave5 were included in the outcome variables, resulting in a clearer causal relationship. Furthermore, this study also found that changes in AIP were not only related to CMD but also independently positively correlated with CMM. Previous studies have also found that an increase in AIP plays a role in promoting the transition from CMD to CMM. A recent study reported that an increase in AIP is an independent predictor of newly diagnosed coronary heart disease in diabetic patients [22], and a secondary data analysis based on the ACCORD study also confirmed this association, with an increase in AIP being closely related to adverse cardiovascular events (including stroke) in diabetic patients [21]. In this study, cumulative AIP was independently positively correlated with CMD, similar to recent research findings. A cohort study involving 54,123 Chinese individuals with an average age of 49.05±11.84 years and a follow-up of 11.3 years found that when the cumulative AIP level was higher than 0.28, the risk of ischemic stroke was 1.45 times higher than when it was lower than -0.50, making cumulative AIP an independent predictor of ischemic stroke [24]. Data from the China Kailuan study also found that cumulative average AIP was an independent predictor of ischemic and hemorrhagic stroke [35].

Subgroup analysis revealed gender differences in the relationship between changes in AIP and CMD, with significant results in men but not in women. The significant interaction effect between cumulative AIP and gender (P for interaction = 0.042) is consistent with previous findings of gender differences in the relationship between AIP and CMD, but this difference is not fixed. Shi et al.'s analysis of NHANES data found that AIP was significantly associated with diabetes and glucose intolerance in women but not in men [36]. However, Zhang et al. [20] reported that an increase in AIP was positively correlated with metabolic syndrome in the Chinese rural population, being significant in male patients but not in females. Cai et al. also found that AIP was an independent risk factor for coronary heart disease in men but not in women [37]. This may be related to differences in lifestyle and genetic background among different regional populations.

The occurrence of CMD is closely related to genetics, environment, and unhealthy lifestyles [8]. Sex differences in the transcriptome may arise from the expression of Y-encoded genes and lead to male-specific cardiovascular phenotypes. In the 2000s, observations linking gene variants on the Y chromosome to hypertension were reported, which could contribute to the higher incidence of CVD in males compared with females [38]. Further results indicated that a locus on the Y chromosome may influence LDL levels, independent of testosterone levels [39]. Notably, it was reported that a severe form of CAD in men was linked to a Y-chromosomal gene variant possibly through interactions of immunity and inflammation [40]. In summary, these data demonstrate that gene variants on the Y chromosome contribute to cardiovascular phenotypes in men. It is proposed that enhanced pro-inflammatory state, associated with HDL dysfunction and autoimmune activation are major determinants of the gender difference in CMD risk [41]. The pro-inflammatory state/oxidative stress, induced by excess adiposity, inflammatory cytokines, circulating Lp(a) and certain enzymes, affect the female to a greater extent. Greater absorption of long-chain fatty acids in women appears a notable contributor to impaired HDL function. The consequences of the pro-inflammatory state, different in one sex, may attenuate the effects of insulin resistance. Furthermore, obesity and proinflammatory states, physiological and psychological stressors, environmental toxins or endocrine disruptors, such as bisphenols, affect sex hormone profiles in men and women and should be included in patient documentation [42]. Lifestyle factors are known to have a strong effect on human phenotypes, which is particularly important as they can be modified to improve health. Studies have reported that smokers have higher AIP levels [43], and the probability of smoking is higher in men than in women, which may explain why men have a higher risk of CMD than women. In addition, research suggests that compared to women, men tend to be both less knowledgeable about nutrition and less concerned about the adverse consequences of unhealthy eating until they have already developed a diet-related health condition [44, 45]. Lifestyle modification, lipid management and blood pressure control are needed for prevention of CMD. Substantial future research is required in regard to identifying the disparity between sexes for the extent of conventional and non-conventional risk factors for CMD.

The linear relationship between cumulative AIP and CMD, with further analysis showing that this phenomenon is mainly reflected in the endpoints of diabetes, with no clear relationship with heart disease and stroke. It is worth noting that cumulative AIP has a non-linear association with stroke in men, while it is linear in the overall population, which may be related to the lack of significant relationship between cumulative AIP and stroke in women. Zhang et al. [35] found that the RCS curve of average AIP and stroke risk in the Chinese general population changed to a "J-shaped" curve. Shi et al. [36] found that AIP had a linear relationship with prediabetes or diabetes.

The mechanism by which AIP is associated with the occurrence of CMD is not fully understood, but the dyslipidemia, atherosclerosis, and insulin resistance reflected by AIP may be the main mechanisms of CMD onset [20]. From the formula, it can be seen that an increase in AIP mainly depends on an increase in TG or a decrease in HDL-C. An increase in TG can accelerate the oxidation of LDL-C, cause endothelial dysfunction, and promote the occurrence of atherosclerosis [46], while HDL-C can antagonize the occurrence of atherosclerosis. Therefore, the imbalance between TG and HDL-C may lead to atherosclerosis, which is a recognized mechanism of stroke and heart disease. In addition, high levels of TG in plasma may reduce the number and activity of insulin receptors on adipocytes and prevent insulin from binding to receptors by competing with glucose for entry into cells, leading to diabetes. A decrease in HDL-C levels may also lead to a decrease in insulin secretion and sensitivity, causing IR. IR will affect the liver's metabolism of sugar and fat, causing plasma TG levels to rise while lowering HDL-C levels [47]. This association can be explained by a "vicious cycle" [48, 49].

The strengths of this study include: 1) The use of a scientific machine learning method (K-means clustering) to classify changes in AIP and explore the relationship between changes in AIP and two years of exposure (cumulative AIP) with CMD, finding significant effects of changes in AIP and increased cumulative AIP on CMD in men, thus compensating for deficiencies in previous studies to some extent; 2) The use of various sensitivity analysis methods to test the stability of the results, yielding conclusions with high credibility; 3) The data source is CHARLS, a cohort study that conducts long-term follow-up of the elderly in China, with high data reliability.

However, this study also has some limitations: 1) The measurement of disease was based on self-reporting, which may underestimate the actual prevalence and also cannot distinguish specific types of heart disease; 2) The study subjects were middle-aged and elderly Chinese individuals, and the conclusions may mainly apply to East Asian populations, with applicability to people under 45 years of age still unclear; 3) Although this study found an independent positive relationship between AIP and CMD and CMM, it did not further explore the conversion model between CMD and CMM and the prognosis of CMM patients, which will be the focus of subsequent research.

## Conclusion

Persistently high AIP levels, as well as increases or decreases from the median level, can increase the risk of CMD in middle-aged and elderly Chinese men. Actively controlling AIP levels can help reduce the risk of CMD, and early detection and control of blood lipid levels are of great significance for the prevention and treatment of CMD. However, given the relatively small sample size of this study, more multicenter, large-sample cohort studies are needed in the future to further explore the relationship between AIP and CMD.

### Supplementary Information

**Additional file 1:** **Table s1. **Comparison of Missing Values and Complete Dataset Baseline Characteristics. Continuous variables are expressed as mean ± standard deviation or interquartile range, and categorical variables are expressed as frequencies (n) and percentages (%). Abbreviations: BMI = Body Mass Index, TC = Total Cholesterol, TG = Triglyceride, LDL-C = Low-Density Lipoprotein Cholesterol, HDL-C = High-Density Lipoprotein Cholesterol, UA = Uric Acid, GLU = Glucose, Cr = Creatinine, BUN = Bilirubin, HbA1c = Hemoglobin A1c, CRP = C-Reactive Protein, SBP = Systolic Blood Pressure, DBP = Diastolic Blood Pressure, AIP = Atherogenic Index of Plasma. **Table s2a.** Associations between Cumulative AIP and CMD after PSM and removal of variables with missing values. Results are presented as Odds Ratios (OR) with 95% Confidence Intervals (CI). Abbreviations as in Table 2. **Table s2b.** Associations between Changes in AIP and CMD after PSM and removal of variables with missing values. Results are presented as Odds Ratios (OR) with 95% Confidence Intervals (CI). Abbreviations as in Table 2. **Table s3a.** Associations Between Cumulative AIP and CMD Incidence After  Removing Outliers and Multiple Imputation. Results are presented as Odds Ratios (OR) with 95% Confidence Intervals (CI). **Table s3b:** Associations Between Changes in AIP and CMD Incidence After Removing Outliers and Multiple Imputation. Results are presented as Odds Ratios (OR) with 95% Confidence Intervals (CI). Abbreviations as in Table 2.

**Additional file 2. Supplementary Figure. A:** Histograms and probability density plots of AIP2012 for groups class1-4, illustrating the data distribution within these groups. B: Histograms and probability density plots of AIP2015 for groups class1-4, showing the data distribution. C-F: Box plots of paired T-tests between AIP2012 and AIP2015 for groups class1-4. **** =  *p*<0.001

## Data Availability

No datasets were generated or analysed during the current study.

## Abbreviations

IR: Insulin Resistance
CMD: Cardiometabolic Disease
CMM: Cardiometabolic Disease
AIP: Atherogenic Index of Plasma
CHARLS: China Health and Retirement Longitudinal Study
TG: Triglyceride
GLU: Glucose
BMI: Body Mass Index
RCS: Restricted Cubic Spline
SD: Standard Deviation
OR: Odds Ratio
CI: Confidence Interval
SBP: Systolic Blood Pressure
DBP: Diastolic Blood Pressure
HbA1c: Glycosylated Hemoglobin
TC: Total Cholesterol
HDL-C: High-Density Lipoprotein Cholesterol
LDL-C: Low-Density Lipoprotein Cholesterol
CRP: C - reactive protein
UA: Uric Acid
Cr: Creatinine
BUN: Blood Urea Nitrogen
