# Supplementary material for: Gender differences in the association between changes in the atherogenic index of plasma and cardiometabolic diseases: a cohort study
Source: Lipids Health Dis. 2024 May 7;23:135. doi: 10.1186/s12944-024-02117-w (PMC11075304; doi:10.1186/s12944-024-02117-w)
Supplement: Supplementary file 1 — Additional file 1: Table s1. Comparison of Missing Values and Complete Dataset Baseline Characteristics. Continuous variables are expressed as mean ± standard deviation or interquartile range, and categorical variables are expressed as frequencies (n) and percentages (%). Abbreviations: BMI = Body Mass Index, TC = Total Cholesterol, TG = Triglyceride, LDL-C = Low-Density Lipoprotein Cholesterol, HDL-C = High-Density Lipoprotein Cholesterol, UA = Uric Acid, GLU = Glucose, Cr = Creatinine, BUN = Bilirubin, HbA1c = Hemoglobin A1c, CRP = C-Reactive Protein, SBP = Systolic Blood Pressure, DBP = Diastolic Blood Pressure, AIP = Atherogenic Index of Plasma. Table s2a. Associations between Cumulative AIP and CMD after PSM and removal of variables with missing values. Results are presented as Odds Ratios (OR) with 95% Confidence Intervals (CI). Abbreviations as in Table 2. Table s2b. Associations between Changes in AIP and CMD after PSM and removal of variables with missing values. Results are presented as Odds Ratios (OR) with 95% Confidence Intervals (CI). Abbreviations as in Table 2. Table s3a. Associations Between Cumulative AIP and CMD Incidence After Removing Outliers and Multiple Imputation. Results are presented as Odds Ratios (OR) with 95% Confidence Intervals (CI). Table s3b: Associations Between Changes in AIP and CMD Incidence After Removing Outliers and Multiple Imputation. Results are presented as Odds Ratios (OR) with 95% Confidence Intervals (CI). Abbreviations as in Table 2. [file 12944_2024_2117_MOESM1_ESM.docx]

**Table s1 Comparison of Missing Values and Complete Dataset Baseline Characteristics**

| **Characteristic** | **Missing data，N(%)** | **Overall, N = 3,791** | **Group with Missing Values, N =** 960 | **Complete Data Group, N =** 2,831 | **p-value** |
| --- | --- | --- | --- | --- | --- |
| **age（year）** |  | 57.41±8.37 | 57.55±8.57 | 57.37±8.30 | 0.555 |
| **Sex(male)** |  | 1,773 (47%) | 461 (48%) | 1,312 (46%) | 0.368 |
| **Education** |  |  |  |  | 0.676 |
| low level |  | 3,456 (91%) | 872 (91%) | 2,584 (91%) |  |
| high level |  | 335 (8.8%) | 88 (9.2%) | 247 (8.7%) |  |
| **Current marital status(married/cohabiting)** |  | 3,467 (91%) | 871 (91%) | 2,596 (92%) | 0.353 |
| Residence （rural） |  | 2,596 (68%) | 626 (65%) | 1,970 (70%) | 0.012 |
| **Drinking** |  | 1,491 (39%) | 381 (40%) | 1,110 (39%) | 0.793 |
| **Smoking** |  | 1,460 (39%) | 387 (40%) | 1,073 (38%) | 0.185 |
| **Hypertension** | 346(9.1%) | 1,174 (34%) | 273 (44%) | 901 (32%) | <0.001 |
| **Dyslipidemia** |  | 228 (6.0%) | 69 (7.2%) | 159 (5.6%) | 0.070 |
| **Consumption (ten thousand yuan per year)** | 539(13.6%) | 0.66±0.93 | 0.77±1.23 | 0.65±0.88 | 0.018 |
| **BMI (kg/m2)** | 441(11.6%) | 23.14±3.69 | 23.03±3.67 | 23.16±3.70 | 0.441 |
| **SBP（mmHg）** | 425(11.2%) | 126.28±20.06 | 127.01±19.67 | 126.14±20.13 | 0.893 |
| **DBP（mmHg）** | 426(11.2%) | 74.20±11.85 | 74.26±11.49 | 74.19±11.92 | 0.352 |
| **TC（mg/dl）** |  | 191.16±37.93 | 189.15±40.67 | 191.84±36.94 | 0.057 |
| **LDL-C（mg/dl）** | 7(0.1%) | 114.94±33.49 | 113.67±33.27 | 115.37±33.56 | 0.174 |
| **GLU（mg/dl）** |  | 102.85±19.66 | 103.76±21.64 | 102.55±18.93 | 0.009 |
| **HbA1c（%）** |  | 5.09±0.43 | 5.08±0.46 | 5.09±0.42 | 0.704 |
| **CRP（mg/dl）** |  | 2.27±6.78 | 2.13±4.63 | 2.31±7.37 | 0.479 |
| **BUN（mg/dl）** |  | 15.70±4.38 | 15.67±4.45 | 15.70±4.36 | 0.827 |
| **Cr（mg/dl）** | 2(0.05%) | 0.76±0.18 | 0.76±0.18 | 0.77±0.18 | 0.286 |
| **UA（mg/dl）** |  | 4.34±1.20 | 4.39±1.22 | 4.33±1.19 | 0.137 |
| **TG2012（mg/dl）** |  | 100.00 (71.68, 144.26) | 100.45 (72.57, 144.26) | 99.12 (71.68, 144.26) | 0.018 |
| **HDL-C2012（mg/dl）** |  | 52.10±14.94 | 49.87 (40.98, 59.92) | 50.64 (41.75, 61.08) | 0.019 |
| **TG 2015** |  | 108.85 (79.65, 158.41) | 109.73 (79.65, 157.52) | 107.96 (79.65, 158.41) | 0.681 |
| **HDL-C2015** |  | 52.32±11.80 | 51.99±11.93 | 52.43±11.75 | 0.323 |
| **AIP 2012** |  | 0.32±0.32 | 0.34±0.34 | 0.32±0.32 | 0.085 |
| **AIP 2015** |  | 0.35±0.27 | 0.36±0.27 | 0.35±0.27 | 0.085 |
| **Cumulative AIP** |  | 1.01±0.80 | 1.04±0.82 | 1.00±0.79 | 0.671 |
| **CMD** |  | 918 (24%) | 272 (28%) | 646 (23%) | <0.001 |

**Table s2a Associations between Cumulative AIP and CMD after PSM and removal of variables with missing values**

|  | **Original Data Model** | | | **Model Excluding Any Variables with Missing Values** | | | **Model After Propensity Score Matching** | | |
| --- | --- | --- | --- | --- | --- | --- | --- | --- | --- |
| **Characteristic** | **OR** | **95% CI** | **p-value** | **OR** | **95% CI** | **p-value** | **OR** | **95% CI** | **p-value** |
| **Cumulative AIP** | 1.15 | (1.02- 1.29) | 0.018 | 1.19 | (1.07-1.31) | <0.001 | 1.18 | (1.03-1.36) | 0.016 |
| **age** | 1.02 | (1.01- 1.03) | <0.001 | 1.03 | (1.02-1.04) | <0.001 | 1.02 | (1.01-1.03) | 0.005 |
| **Sex** | 1.08 | (0.75-1.34) | 0.993 | 1.02 | (0.81-1.31) | 0.858 | 0.99 | (0.71-1.40) | 0.971 |
| **Education** | 1.09 | (0.78-1.49) | 0.621 | 1.04 | (0.78-1.37) | 0.773 | 1.13 | (0.77-1.62) | 0.529 |
| **BMI** | 1.02 | (1.00-1.05) | 0.068 |  |  |  | 1.03 | (0.99-1.06) | 0.103 |
| **Current marital status(married/cohabiting)** | 0.95 | (0.69-1.30) | 0.732 | 0.99 | (0.75-1.30) | 0.911 | 1.03 | (0.72-1.50) | 0.881 |
| Residence （rural） | 1.06 | (0.87-1.29) | 0.550 | 1.09 | (0.92-1.29) | 0.324 | 1.06 | (0.85-1.33) | 0.619 |
| **Drinking** | 1.04 | (0.84-1.29) | 0.736 | 1.07 | (0.89-1.28) | 0.486 | 1.04 | (0.83-1.31) | 0.721 |
| **Smoking** | 0.83 | (0.64-1.08) | 0.158 | 0.88 | (0.70- 1.09) | 0.238 | 0.98 | (0.77-1.26) | 0.965 |
| **Hypertension** | 1.46 | (1.13-1.89) | 0.004 |  |  |  | 1.35 | (1.00-1.82) | 0.047 |
| **Dyslipidemia** | 1.83 | (1.29-2.56) | <0.001 | 2.02 | (1.52-2.68) | <0.001 | 0.93 | (1.28-2.90) | 0.002 |
| **Consumption** | 1.06 | (0.96-1.17) | 0.214 |  |  |  | 1.01 | (0.89-1.13) | 0.856 |
| **BUN** | 1.00 | (0.98-1.02) | 0.828 | 1.00 | (0.98- 1.02) | 0.887 | 0.99 | (0.96-1.01) | 0.359 |
| **Cr** | 1.03 | (0.54-1.96) | 0.921 |  |  |  | 0.92 | (0.43-1.93) | 0.831 |
| **UA** | 1.02 | (0.93-1.12) | 0.673 | 1.03 | (0.96- 1.10) | 0.456 | 1.02 | (0.92-1.13) | 0.696 |
| **TC** | 1.00 | (1.00, 1.00) | 0.882 | 1.00 | (1.00- 1.00) | 0.012 | 1.00 | (1.00-1.01) | 0.481 |
| **LDL-C** | 1.00 | (1.00- 1.01) | 0.205 |  |  |  | 1.00 | (1.0-1.01) | 0.803 |
| **CRP** | 1.00 | (0.99-1.01) | 0.492 | 1.00 | (0.99-1.01) | 0.742 | 1.01 | (0.99-1.02) | 0.462 |
| **GLU** | 1.00 | (1.00- 1.01) | 0.731 | 1.00 | (1.00-1.01) | 0.518 | 1.00 | (1.00-1.01) | 0.420 |
| **HbA1c** | 1.05 | (0.85-1.30) | 0.639 | 1.10 | (0.92-1.31) | 0.212 | 1.03 | (0.80-1.33) | 0.795 |
| **SBP** | 1.00 | (0.99- 1.00) | 0.498 |  |  |  | 1.00 | (1.00, 1.01) | 0.466 |
| **No. Obs.** | 2,875 |  |  | 3,791 |  |  | 2,072 |  |  |
| **AIC** | 3,115 |  |  | 4,135 |  |  | 2,319 |  |  |
| **BIC** | 3,246 |  |  | 4,235 |  |  | 2,443 |  |  |

**Table s2b Associations between Changes in AIP and CMD after PSM and removal of variables with missing values**

|  | **Original Data Model** | | | **Model Excluding Any Variables with Missing Values** | | | **Model After Propensity Score Matching** | | |
| --- | --- | --- | --- | --- | --- | --- | --- | --- | --- |
| **Characteristic** | **OR** | **95% CI** | **p-value** | **OR** | **95% CI** | **p-value** | **OR** | **95% CI** | **p-value** |
| **class** |  |  |  |  |  |  |  |  |  |
| **class 1** | — | — |  | — | — |  | — | — |  |
| **class 2** | 1.31 | (1.04-1.66) | 0.021 | 1.26 | (1.03-1.54) | 0.022 | 1.55 | (1.18-2.04) | 0.002 |
| **class 3** | 1.34 | (1.04-1.71) | 0.023 | 1.40 | (1.14-1.73) | 0.001 | 1.41 | (1.06-1.88) | 0.019 |
| **class 4** | 1.40 | (1.04-1.88) | 0.026 | 1.46 | (1.13-1.86) | 0.002 | 1.52 | (1.09-2.13) | 0.014 |
| **Categories** |  |  |  |  |  |  |  |  |  |
| **Q1[-1.65,0.449]** | — | — |  | — | — |  | — | — |  |
| **Q2(0.449,0.937]** | 1.13 | (0.87-1.47) | 0.361 | 1.17 | (0.94-1.47) | 0.158 | 1.40 | (1.02-1.93) | 0.040 |
| **Q3(0.937,1.49]** | 1.41 | (1.09-1.83) | 0.010 | 1.51 | (1.21-1.88) | <0.001 | 1.68 | (1.24-2.29) | <0.001 |
| **Q4(1.49,5.36]** | 1.28 | (0.98-1.69) | 0.070 | 1.44 | (1.16-1.82) | 0.001 | 1.46 | (1.07-2.00) | 0.018 |
| **Q1-Q2** |  |  |  |  |  |  | - | - | - |
| **Q3-Q4** |  |  |  |  |  |  | 1.21 | (1.00-1.49） | 0.050 |
| No. Obs. | 2,875 |  |  | 3,791 |  |  | 2,072 |  |  |
| **AIC** | 3,115 |  |  | 4,135 |  |  | 2,319 |  |  |
| **BIC** | 3,246 |  |  | 4,235 |  |  | 2,443 |  |  |

**Table s3a Associations Between Cumulative AIP and CMD Incidence After Removing Outliers and Multiple Imputation**

|  | **Original Data Model** | | | **Model After Removing Outliers** | | | **Model After Multiple Imputation** | | |
| --- | --- | --- | --- | --- | --- | --- | --- | --- | --- |
| **Characteristic** | **OR** | **95% CI** | **p-value** | **OR** | **95% CI** | **p-value** | **OR** | **95% CI** | **p-value** |
| **Cumulative AIP** | 1.15 | (1.02- 1.29) | 0.018 | 1.14 | (1.01-1.29) | 0.043 | 1.15 | (1.03-1.29) | 0.010 |
| **age** | 1.02 | (1.01- 1.03) | <0.001 | 1.02 | (1.01- 1.03) | <0.001 | 1.03 | (1.02-1.04) | <0.001 |
| **Sex** | 1.08 | (0.75-1.34) | 0.993 | 1.00 | (0.74-1.34) | 0.981 | 0.98 | (0.76-1.26) | 0.872 |
| **Education** | 1.09 | (0.78-1.49) | 0.621 | 1.10 | (0.79-1.51) | 0.574 | 1.02 | (0.77-1.36) | 0.865 |
| **BMI** | 1.02 | (1.00-1.05) | 0.068 | 1.02 | (1.00-1.05) | 0.078 | 1.03 | (1.00-1.05) | 0.044 |
| **Current marital status(married/cohabiting)** | 0.95 | (0.69-1.30) | 0.732 | 0.94 | (0.69-1.29) | 0.685 | 1.01 | (0.77-1.33) | 0.934 |
| **Residence （rural）** | 1.06 | (0.87-1.29) | 0.550 | 1.07 | (0.88-1.30) | 0.552 | 1.14 | (0.96-1.35) | 0.141 |
| **Drinking** | 1.04 | (0.84-1.29) | 0.736 | 1.04 | (0.83-1.29) | 0.747 | 1.06 | (0.88-1.28) | 0.509 |
| **Smoking** | 0.83 | (0.64-1.08) | 0.158 | 0.82 | (0.63-1.06) | 0.131 | 0.89 | (0.71-1.11) | 0.297 |
| **Hypertension** | 1.46 | (1.13-1.89) | 0.004 | 1.46 | (1.13-1.89) | 0.004 | 1.31 | (1.03-1.67) | 0.028 |
| **Dyslipidemia** | 1.83 | (1.29-2.56) | <0.001 | 1.84 | (1.30-2.58) | <0.001 | 1.84 | (1.38-2.45) | <0.001 |
| **Consumption** | 1.06 | (0.96-1.17) | 0.214 | 1.07 | (0.97-1.17) | 0.186 | 1.03 | (0.95-1.12) | 0.484 |
| **BUN** | 1.00 | (0.98-1.02) | 0.828 | 1.00 | (0.98-1.02) | 0.184 | 1.00 | (0.98-1.02) | 0.728 |
| **Cr** | 1.03 | (0.54-1.96) | 0.921 | 1.02 | (0.53-1.95) | 0.944 | 0.90 | (0.52-1.57) | 0.712 |
| **UA** | 1.02 | (0.93-1.12) | 0.673 | 1.02 | (0.93-1.12) | 0.663 | 1.01 | (0.94-1.09) | 0.765 |
| **TC** | 1.00 | (1.00, 1.00) | 0.882 | 1.00 | (0.99-1.00) | 0.785 | 1.00 | (1.00-1.01) | 0.872 |
| **LDL-C** | 1.00 | (1.00- 1.01) | 0.205 | 1.00 | (1.00- 1.01) | 0.203 | 1.00 | (1.00-1.01) | 0.055 |
| **CRP** | 1.00 | (0.99-1.01) | 0.492 | 1.00 | (0.99-1.01) | 0.496 | 1.00 | (0.99-1.01) | 0.818 |
| **GLU** | 1.00 | (1.00- 1.01) | 0.731 | 1.00 | (1.00- 1.01) | 0.900 | 1.00 | (1.00-1.01) | 0.405 |
| **HbA1c** | 1.05 | (0.85-1.30) | 0.639 | 1.05 | (0.85-1.30) | 0.556 | 1.07 | (0.90-1.29) | 0.386 |
| **SBP** | 1.00 | (0.99- 1.00) | 0.498 | 1.00 | (0.99- 1.00) | 0.465 | 1.01 | (1.00-1.01) | 0.495 |
| **No. Obs.** | 2,875 |  |  |  | 2844 |  | 3791 |  |  |
| **AIC** | 3,115 |  |  |  |  |  |  |  |  |
| **BIC** | 3,246 |  |  |  |  |  |  |  |  |

**Table s3b Associations Between Changes in AIP and CMD Incidence After Removing Outliers and Multiple Imputation**

|  | **Original Data Model** | | | **Model After Removing Outliers** | | | **Model After Multiple Imputation** | | |
| --- | --- | --- | --- | --- | --- | --- | --- | --- | --- |
| **Characteristic** | **OR** | **95% CI** | **p-value** | **OR** | **95% CI** | **p-value** | **OR** | **95% CI** | **p-value** |
| **class** |  |  |  |  |  |  |  |  |  |
| **class 1** | — | — |  |  |  |  | — | — | — |
| **class 2** | 1.31 | (1.04-1.66) | 0.021 | 1.31 | (1.04-1.66) | 0.022 | 1.18 | (0.97-1.45) | 0.106 |
| **class 3** | 1.34 | (1.04-1.71) | 0.023 | 1.33 | (1.04-1.71) | 0.025 | 1.27 | (1.02-1.58) | 0.029 |
| **class 4** | 1.40 | (1.04-1.88) | 0.026 | 1.39 | (1.03-1.87) | 0.029 | 1.34 | (1.04-1.74) | 0.026 |
| **Categories** |  |  |  |  |  |  |  |  |  |
| **Q1[-1.65,0.449]** | — | — |  |  |  |  | — | — |  |
| **Q2(0.449,0.937]** | 1.13 | (0.87-1.47) | 0.361 | 1.12 | (0.86-1.46) | 0.386 | 1.11 | (0.88-1.39) | 0.388 |
| **Q3(0.937,1.49]** | 1.41 | (1.09-1.83) | 0.010 | 1.40 | (1.08-1.82) | 0.011 | 1.38 | (1.10-1.73) | 0.005 |
| **Q4(1.49,5.36]** | 1.28 | (0.98-1.69) | 0.070 | 1.28 | (0.97-1.68) | 0.076 | 1.29 | (1.02-1.63) | 0.035 |
| **No. Obs.** | 2,875 |  |  |  | 2844 |  | 3791 |  |  |
|  |  |  |  |  |  |  |  |  |  |

**Table S4: Associations Between Different Categories of Change in AIP and the Incidence of CMD, Stratified by Gender**

| **Subgroups** | **N** | **Q1[-1.65,0.449]** | **Q2(0.449,0.937]** | **Q3(0.937,1.49]** | **Q4(1.49,5.36]** | ***P for trend*** | ***P for ineraction*** |
| --- | --- | --- | --- | --- | --- | --- | --- |
|  |  |  | ***OR(95% CI)*** | ***OR(95% CI)*** | ***OR(95% CI)*** |  |  |
| **Sex** |  |  |  |  |  |  | 0.301 |
| **male** | **1773** | ref | 1.30(0.88-1.92) | 1.75(1.19, 2.59) | 1.60(1.06-2.40) | 0.010 |  |
| **female** | **2018** | ref | 0.97(0.68-1.39) | 1.14(0.80-1.64) | 1.05(0.73-1.53) | 0.312 |  |
| Adjustments were made for age, education , current marital status, residence , consumption, smoking, drinking,BMI,SBP, hypertension, dyslipidemia, TC, LDL-C, GLU, HbA1c, CRP, Cr, BUN, UA. | | | | | | | |
