# Supplementary figures and images for: Gender differences in the association between changes in the atherogenic index of plasma and cardiometabolic diseases: a cohort study
Source: Lipids Health Dis. 2024 May 7;23:135. doi: 10.1186/s12944-024-02117-w (PMC11075304; doi:10.1186/s12944-024-02117-w)

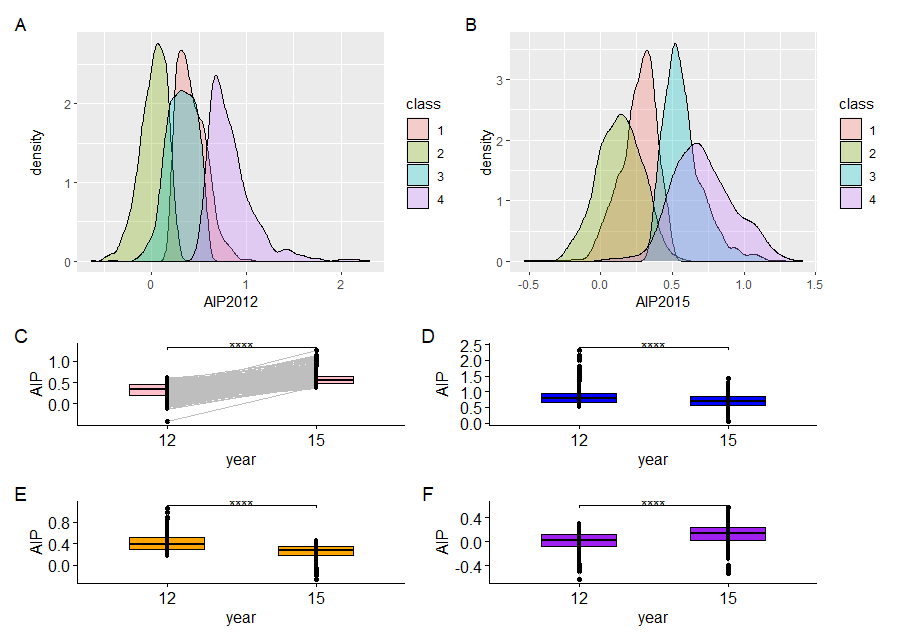

Supplement: Supplementary file 2 — Additional file 2. Supplementary Figure. A: Histograms and probability density plots of AIP2012 for groups class1-4, illustrating the data distribution within these groups. B: Histograms and probability density plots of AIP2015 for groups class1-4, showing the data distribution. C-F: Box plots of paired T-tests between AIP2012 and AIP2015 for groups class1-4. **** = p<0.001 [file 12944_2024_2117_MOESM2_ESM.tiff]
